# A real‐world data of Immune checkpoint inhibitors in solid tumors from India

**DOI:** 10.1002/cam4.3617

**Published:** 2021-02-16

**Authors:** Vanita Noronha, George Abraham, Vijay Patil, Amit Joshi, Nandini Menon, Abhishek Mahajan, Amit Janu, Srushti Jain, Vikas T Talreja, Akhil Kapoor, Gunjesh Kumar Singh, Satvik Khaddar, Kushal Gupta, Narmadha Rathinasamy, Sujay Srinivas, Amit Agrawal, Pradeep Ventrapati, Kumar Prabhash

**Affiliations:** ^1^ Department of Medical Oncology Tata Memorial Hospital Mumbai India; ^2^ Department of Radiodiagnosis Tata Memorial Hospital Mumbai India

**Keywords:** checkpoint inhibitors, CPI, hepatitis, Immunotherapy, India, irAE, Nivolumab, Pembrolizumab, pneumonitis

## Abstract

**Background:**

Checkpoint inhibitors (Nivolumab and Pembrolizumab) are approved for multiple indications in solid tumors. However access to these therapies is limited in low and middle income countries. Hence we performed an audit to identify accessibility, adverse event rates, compliance, progression free survival and overall survival in solid tumors.

**Methods:**

This was a single center retrospective analysis of prospective data base of patients with non‐melanoma solid tumors who were treated with immunotherapy from August 2015 to November 2018. Adverse events during immunotherapy were documented and graded using CTCAE (Common terminology criteria for adverse events), v. 4.02. The response rates to immunotherapy, toxicities and the time to onset and resolution of toxicities were also evaluated as secondary endpoints.

**Results:**

Out of 9610 patients, only 155 patients (1.61%) could receive immunotherapy. The most common malignancies included metastatic non‐small cell lung cancer, metastatic renal cell carcinoma, metastatic urothelial carcinoma and relapsed/recurrent head and neck squamous cell carcinoma. Median overall survival in patients who received immunotherapy in non‐melanoma solid malignancies was 5.37 months (95% CI, 3.73–9.73). Poor performance status at baseline was the only adverse prognostic factor. The median progression free survival was 2.57 months (95% CI, 1.73–3.83). Immunotherapy was well tolerated with most common side effects being fatigue 14.8% and anorexia 5.8%. The cumulative incidence of immune related adverse events like hepatitis, pneumonitis, colitis and nephritis was less than 10%.

**Conclusion:**

Real‐world data in Indian setting confirms the benefit of immunotherapy in patients with advanced non‐melanoma solid tumors.

## Graphical abstract

This is the first and largest real world data of immunotherapy from India. It focuses on efficacy and tolerability of immune check point inhibitors in Indian patients.

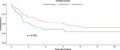

## 1 BACKGROUND

Various forms of immunotherapy especially immune checkpoint inhibitors (ICI) have changed the treatment paradigm of many cancers with poor prognosis in the last decade.^1^, ^2^ These ICI have been approved in malignant melanoma as adjuvant treatment and in metastatic settings,^3^ non‐small cell lung cancer (NSCLC),^4^ renal cell carcinoma,^5^ urothelial carcinoma,^6^ recurrent and metastatic head and neck squamous cell carcinomas,^7^ relapsed and refractory classical Hodgkin's lymphoma ^8^ and microsatellite instability high histology agnostic cancers, gastric^9^ and colorectal cancers.^10^

Immune checkpoint inhibitors (ICI) have been shown to be more effective than the existing standard chemotherapy and are also safer than the chemotherapy in many tumors especially in hepatitis B/ hepatitis C infections and human immune deficiency virus (HIV) infections.^11^, ^12^ Immunotherapy has also fulfilled the unmet need in situations where chemotherapy cannot be used like patients with bladder cancer who are cisplatin ineligible.^13^ Use of ICI extends beyond the chemotherapy especially in patients with poor performance status (PS).^14^ ICI can also be used safely in patients with multiple comorbidities, human immunodeficiency virus infection (HIV), influenza and Severe Acute respiratory syndrome Corona virus‐2 (SARS COVID‐19) infections.^15^ Asian countries have large populations with large opportunities for prospective and retrospective studies as a part of drug development.^16^ We have gradually learned that many drugs like ICI have a differential activity and toxicity spectrum in different ethnic populations.^17^ It is also important to confirm the trial data in the real‐life scenario. Immune checkpoint inhibitors have been available in India since 2015 and their use has increased exponentially over the last 4 years. Hence there is an urgent need to have real‐world data on immunotherapy from low‐ and middle‐income countries like India to understand the impact of the treatment in the clinic. This will help us understand the utility of immunotherapy in routine clinical practice in real‐world settings. Hence we have audited our practice.

## 2 METHODS

This is a single center retrospective study of patients who received nivolumab and pembrolizumab from August 2015 to November 2018. Data censoring was done on 9 February 2019. We retrieved the details of the patients who received checkpoint inhibitors in our hospital from the Solid tumor unit. The data were entered in excel sheet and included the baseline demographic data, comorbid conditions, previous lines of therapies received till progression, duration of each previous line of therapy and their best response, type of PD‐1 inhibitor used (nivolumab/ pembrolizumab) and dose and schedule of nivolumab and pembrolizumab. Response assessment was performed using radiological assessment, and responses were classified according to the Response Evaluation Criteria in Solid Tumors (RECIST) version 1.1. Response assessment was done 2 months after the commencement of ICI or at any symptoms/signs of clinical progression whichever was earlier. Adverse events during immunotherapy were documented and graded using the Common Terminology Criteria for Adverse Events (CTCAE), version 4.02. Data collected included the date of start of immunotherapy, date of stopping immunotherapy, reason for stopping immunotherapy, date of progressive disease and date of death. Univariate and multivariate analyses were performed using Cox regression model for the clinicopathological factors thought to have a possible impact on the overall survival.

### 2.1 Inclusion and exclusion criteria

All patients with recurrent or metastatic cancers (solid tumors) with approved indications for use of ICI were selected for the study. The analysis was done in patients who had received at least 1 cycle of ICI. Pregnant patients and pediatric (<15 years) population were excluded. Among the 9610 patients screened who had approved indications of ICI, 155 (1.6%) patients had received immune checkpoint inhibitors and were analyzed.

### 2.2 Adverse event recording (AER)

At the start of ICI, patients were counseled for the possible toxicities by the treating physician. Patients were subsequently followed up in outpatient department 1 week post the first cycle followed by prior to each ICI cycle which was every 2 weekly for nivolumab and every 3 weekly for pembrolizumab. For any complications, the patients were followed up in emergency department.

The baseline work up prior to start of ICI included complete blood counts, liver and renal function tests, electrocardiogram (ECG), echocardiogram, urine routine and microscopy with proteinuria assessment, hormonal profile including thyroid profile, cortisol and adrenocorticotrophic hormone and estrogen/testosterone. Baseline pulmonary function tests were also done in fit and cooperative patients.

Most common antibiotics used were beta‐lactams like cefoperazone‐sulbactam (50 mg/kg) with maximum dose of 3 g every 12 hourly, aminoglycosides like amikacin at 15 mg/kg every 24 hours, levofloxacin 500 mg once daily. Carbapenem antibiotics like meropenem 50 mg/kg every 8 hourly and colistin 9 million IU loading dose followed by 4.5 million IU every 12 hourly were mostly used as second line or upfront in case of septic shock or inotrope requirement at presentation. Stoppage of antibiotics was based on clinical and radiological response and microbiological culture negativity based on standard institutional guidelines.

### 2.3 Statistics

Descriptive statistics were used for demographic data. Response rate and side effects were calculated in percentages. Overall survival (OS) was defined as the interval between the date of start of the immunotherapeutic agent till the date of death due to any cause or date of last follow‐up. Progression free survival (PFS) was defined as the interval from the date of starting immunotherapeutic agent till the date of progression or death due to any cause if it happened before disease progression or the last follow‐up date whichever was earlier. Progression was defined as clinical worsening of symptoms related to the disease or radiological progression as per RECIST v 1.1 or death due to any cause.

Statistical analysis was done using SPSS software version 22, RStudio version 3.5.2 (for cumulative incidence of toxicities). Time to event analysis was plotted on Kaplan–Meier curves and hazard ratio was calculated using Cox regression analysis.

### 2.4 Data accessibility statement

We state that all the datasheets will be made available to the reviewing journal on request.

### 2.5 Ethics statement

All the patients' details were anonymized before the start of data analysis by the investigators and institutional ethics committee approval was obtained prior to the start of the study.

## 3 RESULTS

Among 9610 patients with solid tumors who had clinical indications for commencement of ICI, only 155 (1.61%) could receive ICI. Financial constraint was the most common limiting factor for use of ICI in our setting.

### 3.1 Baseline characteristics

In lung cancer, the patients received immunotherapy in median second line in palliative setting. In adenocarcinoma of lung, 84% (*n* = 50) had received platinum‐based chemotherapy prior the start of immunotherapy and rest received oral tyrosine kinase inhibitor as first line therapy. Table 1.

**TABLE 1:** The baseline characteristics of the patients treated with immunotherapy are shown below.

Variable	Number of patients; *n* = 155 (%)
Site of primary tumor	
Head and neck cancer	48 (31)
Lung cancer	76 (49)
Adenocarcinoma	59
Squamous cell carcinoma	13
Small cell lung cancer	4
Others (Renal cell carcinoma, urothelial carcinoma, malignant mesothelioma)	31 (20)
Median age in years	57.16 (21.79–85.63) 95% CI
Sex	
Male	119 (76.8)
Female	36 (23.2)
ECOG performance status	
0–1	102 (66.7)
2–3	51 (32.0)
Missing data	2 (1.3)
Brain metastasis	13 (8.4)
Immunotherapy received in median which line (overall data).	2nd (Range, 1–9)

In head and neck SCC, 85% (*n* = 42) received ICI in second line and beyond.

Agents used in immunotherapy as shown in Table 2.

**TABLE 2:** The agents used for immunotherapy were nivolumab and pembrolizumab.

Immunotherapy agent	Number of patients, n = 155 (%)
Nivolumab	151 (97.4)
Pembrolizumab	4 (2.6)
Nivolumab dose	
3 mg/kg every 2 weekly	108 (69.7)
240 mg flat dose every 2 weekly	47 (30.3)
Pembrolizumab‐ 200 mg flat dose every 3 weekly	4 (2.6)

**FIGURE 1:**
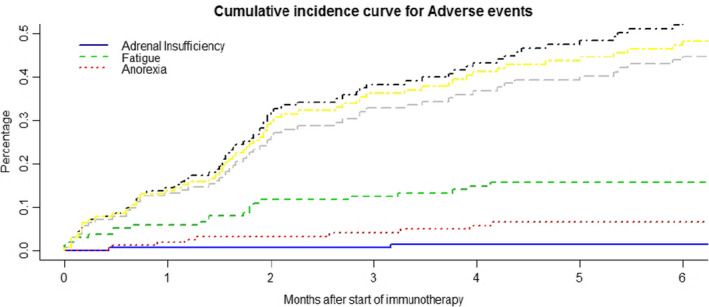
The figure demonstrates the cumulative incidence of adverse events (fatigue, anorexia and adrenal insufficiency) against months after the start of therapy. Competing risk analysis to modify for competing events like death and progression was done and represented in black dotted‐ fatigue, yellow dotted‐ anorexia and grey dotted‐ adrenal insufficiency (RStudio version 3.5.2). The cumulative incidence of these adverse events was below 10% even with consideration of competing events.

**FIGURE 2:**
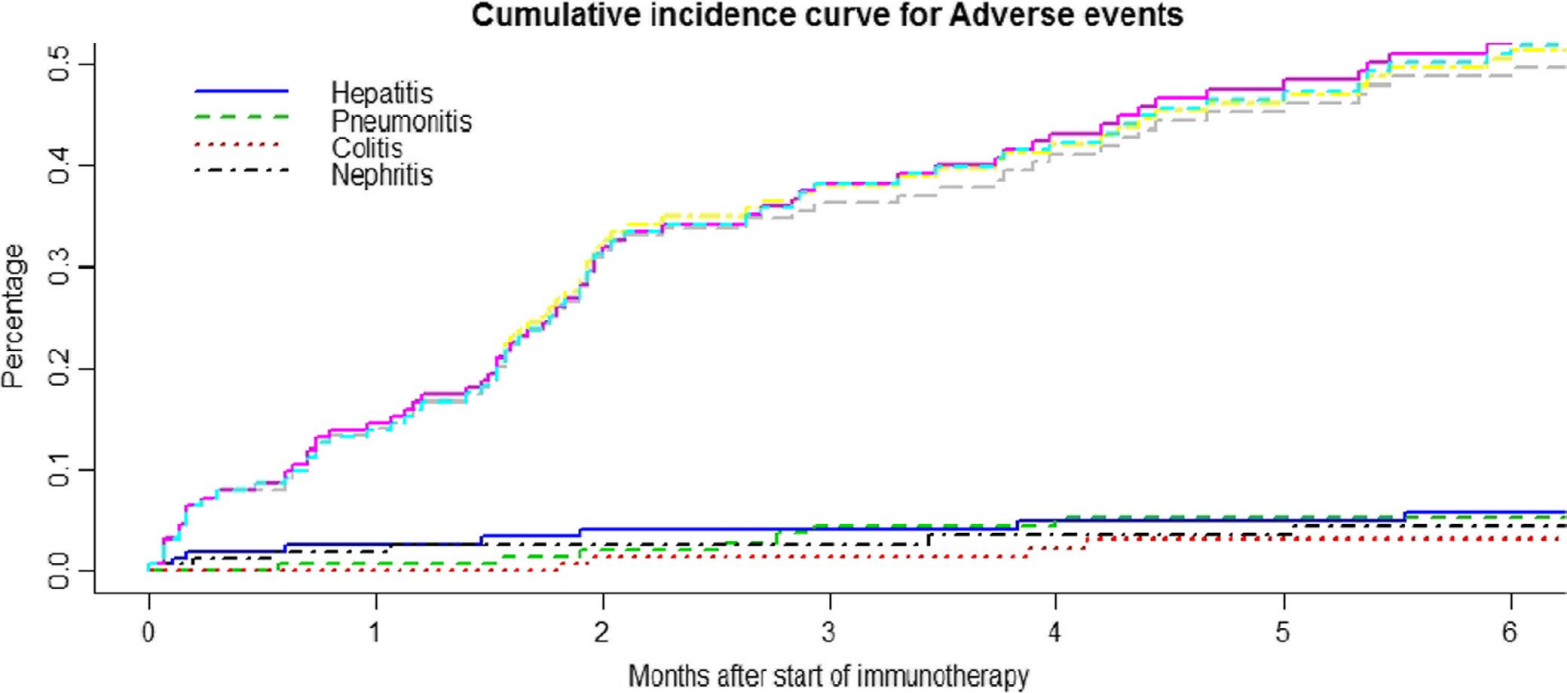
The figure demonstrates the percentage incidence of immune mediated toxicities with time in patients who were treated with immunotherapy. The cumulative incidence of immune mediated toxicities (magenta dotted‐ colitis) was less than 5 percentage after adjusting for competing risk factors like mortality and progression.

The most common adverse events (predominantly grade 1 and 2) associated with immune checkpoint inhibitors were fatigue in 14.8% patients and anorexia in 5.8% patients (Tables 3 and 4). The cumulative incidence of toxicities is shown in Figures 1and 2. It was noted that the incidence of major toxicities were higher in the patients with poor PS (Eastern cooperative oncology group, ECOG 2, 3, 4) 27.5% (*n* = 11/40) when compared to 12.3% (*n* = 13/105) in patients with ECOG 0 and 1.

**TABLE 3:** Adverse events were graded according to Common terminology criteria for adverse events (CTCAE) 4.02.

	Grade 1	Grade 2	Grade 3	Grade 4
Hepatitis	5 (3.2)	0	4 (2.6)	0
Pneumonitis	0	0	4 (2.6)	3 (1.9)
Colitis	2 (1.3)	1 (0.6)	2 (1.3)	0
Nephritis	2 (1.3)	4 (2.6)	0	0
Encephalitis	0	0	1 (0.6)	0
Adrenal insufficiency	3 (1.9)	0	0	0
Thyroiditis	1 (0.6)	0	0	0
Hypophysitis			1 (0.6)	
Hypersensitivity reactions	0	0	0	0
Skin rash	0	3(1.9)	0	0
Nausea and vomiting	4 (2.6)	0	0	0
Mucositis	0	1 (0.6)	1 (0.6)	0
Anorexia	7 (4.5)	2 (1.3)	0	0
Fatigue	11 (7.1)	9 (5.8)	3 (1.9)	0

**TABLE 4:** The time of onset and resolution of adverse events related to immune checkpoint inhibitors are depicted below.

	Median time of onset in months (95% CI)	Median time to resolution in months (95% CI)
Hepatitis	1.47 (0–4.0)	0.6 (0–2)
Pneumonitis	2.53 (0.9–4.2)	24 days (resolved in 2 patients out of 7)
Colitis	3.9 (0–8)	0.8 (0–2.3)
Nephritis	0.5 (0–1.6)	8.8 (1–16.5)
Adrenal insufficiency	3.1 (0–7.5)	Resolved in 1 patient after 120 days
Fatigue	1.4 (0.1–2.6)	5.8 (1.9–10)
Anorexia	1.3 (1.0–1.6)	2.3 (1.4–3.2)

Abbreviations: CI, confidence interval.

### 3.2 Supportive care

**FIGURE 3:**
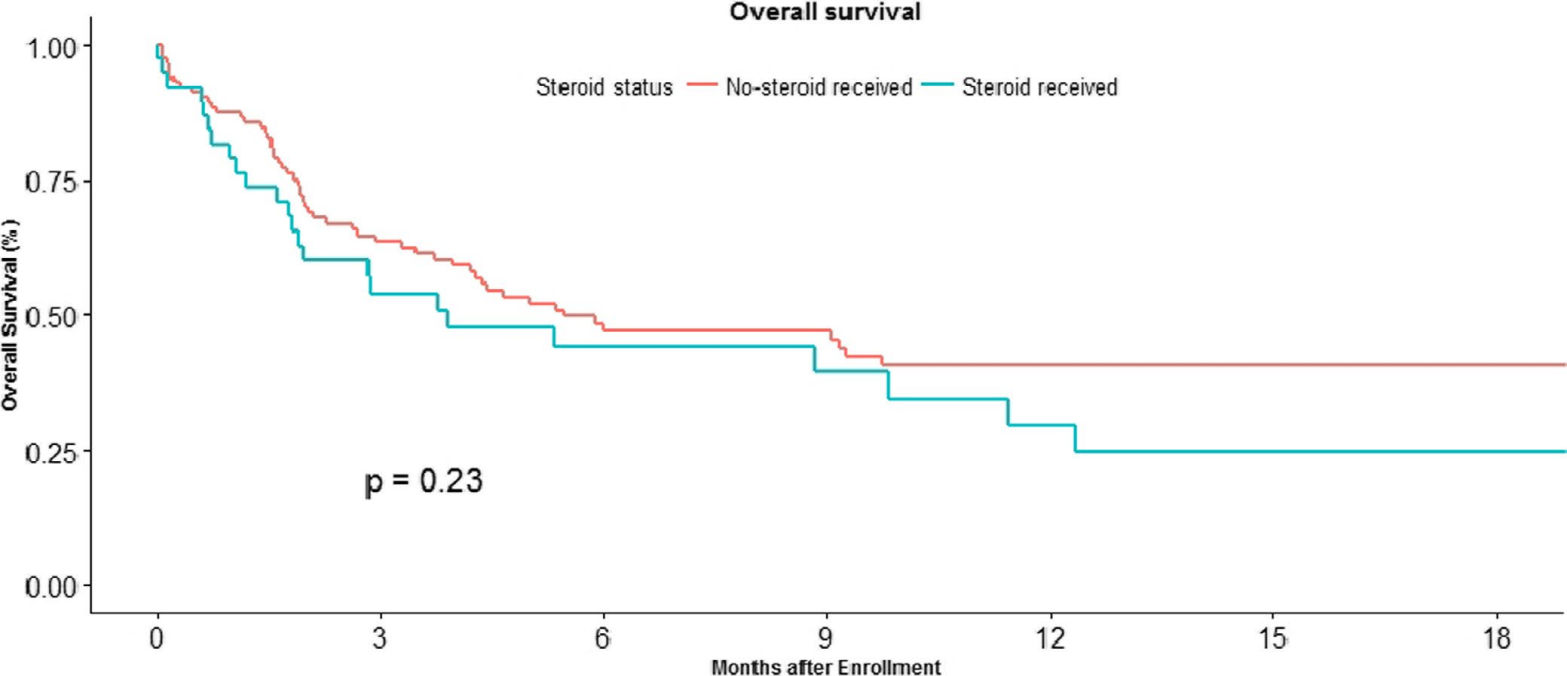
Kaplan Meier curve of overall survival in patients on immune check point inhibitors who have received concomitant steroids (blue) and who did not receive cobcomitant steroids. The median OS in the steroid group was 3.90 (1.80–11.4) months versus 5.47 (3.73‐NA) months in patients who did not receive steroids. (HR‐1.95% CI‐0.963‐1.039). There was no stastically significant difference in overall survival in these groups. *p *= 0.23.

Steroid use of predisolone equivalent of more than 10 mg per day was 24% (38/155). There was no survival advantage in patients who received and did not receive steroids as shown in Figure 3.

**FIGURE 4:**
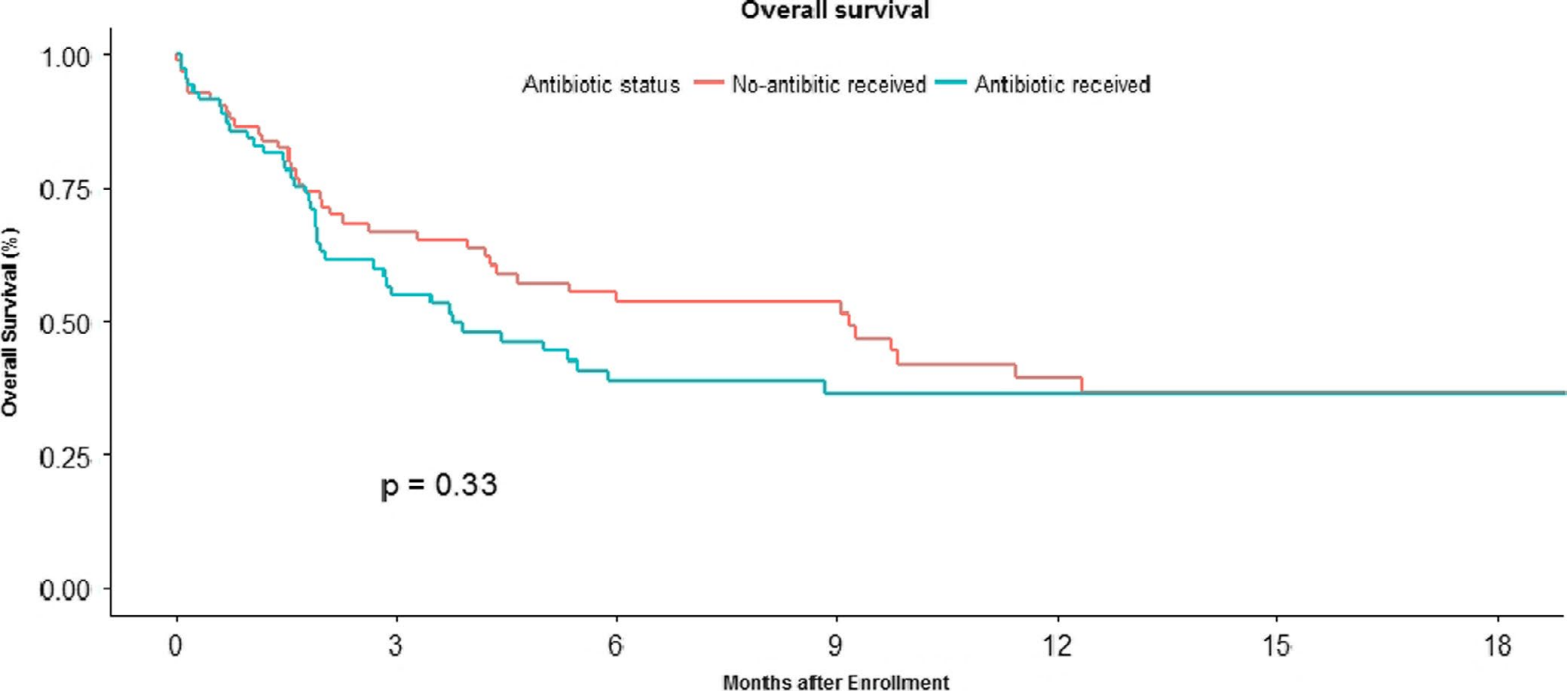
Kaplan Meier curve showing overall survival in patients on immune checkpoint inhibitors who received concomitant antibiotics (included patients with IO who received oral /IV antibiotics for various indications for atleast 5 days) versus patient who did not receive concomitant antibiotics. The median OS in the patients who received antibiotics was 3.90 (1.80–11.4) when compared to 9.17 (4.20–12.33) in patients who did not receive antibiotics with a trend to significance with *p* value‐ 0.053. (HR‐1.023; 95% CI 22121–1.1047).

Use of concomitant antibiotics and steroids were as per current hospital policies and guidelines and were shown not to have an impact on overall survival (Figure 4). The choice of antibiotics was based on clinical and radiological focus for infection at presentation and subsequently modified based on response and culture reports. Requirement of antibiotics (oral or IV) was 44% (70/155). The median duration of antibiotic use was 10 (5–40) days.

## 4 OUTCOMES

The median duration of follow‐up was 2 (0.5–21) months.

### 4.1 Response assessment

The response assessment was done as per RECIST and imaging of the primary site with sites of metastasis was carried out at baseline before the start of ICI and every 2 months. The response rate was 19.4% (complete response +partial response)(Table 5).

**TABLE 5:** The response rate of patients treated with ICI in non‐melanoma solid tumors is shown below.

Response	Number (%)
Complete response	2 (1.3)
Partial response	28 (18.1)
Stable disease	37 (23.9)
Progressive disease	52 (33.5)
Mixed response	2 (1.3)
First response assessment pending at date of analysis)	34 (21.9)

### 4.2 Progression free survival (PFS)

**FIGURE 5:**
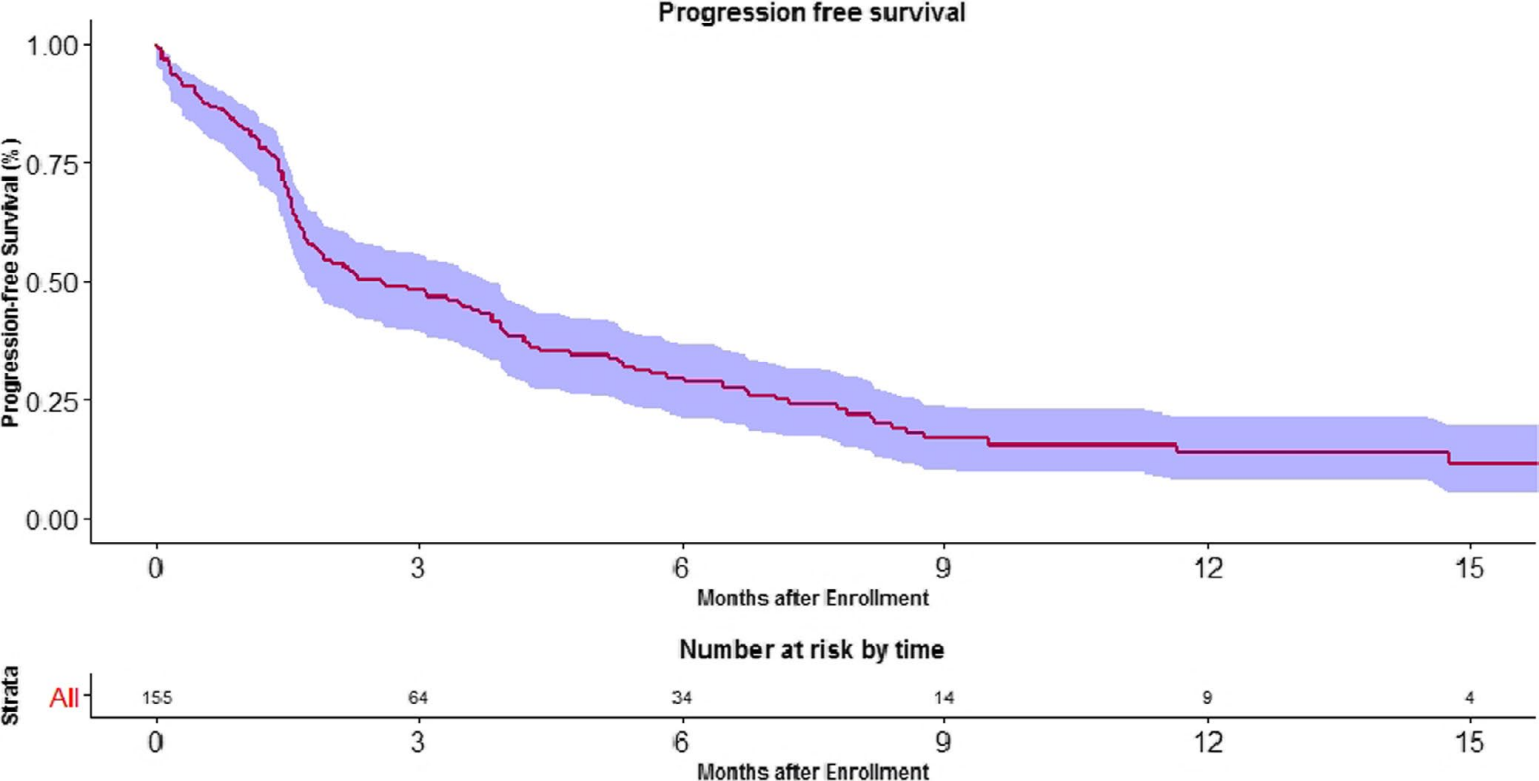
Kaplan Meier curve showing the progression free survival in patients treated with ICI. The 6 month progression free survival was 25.2%.

The median PFS of immunotherapy among non‐melanoma solid cancers was 2.57 months (95% CI, 1.73–3.83) (Figure 5).

### 4.3 Overall survival

**FIGURE 6:**
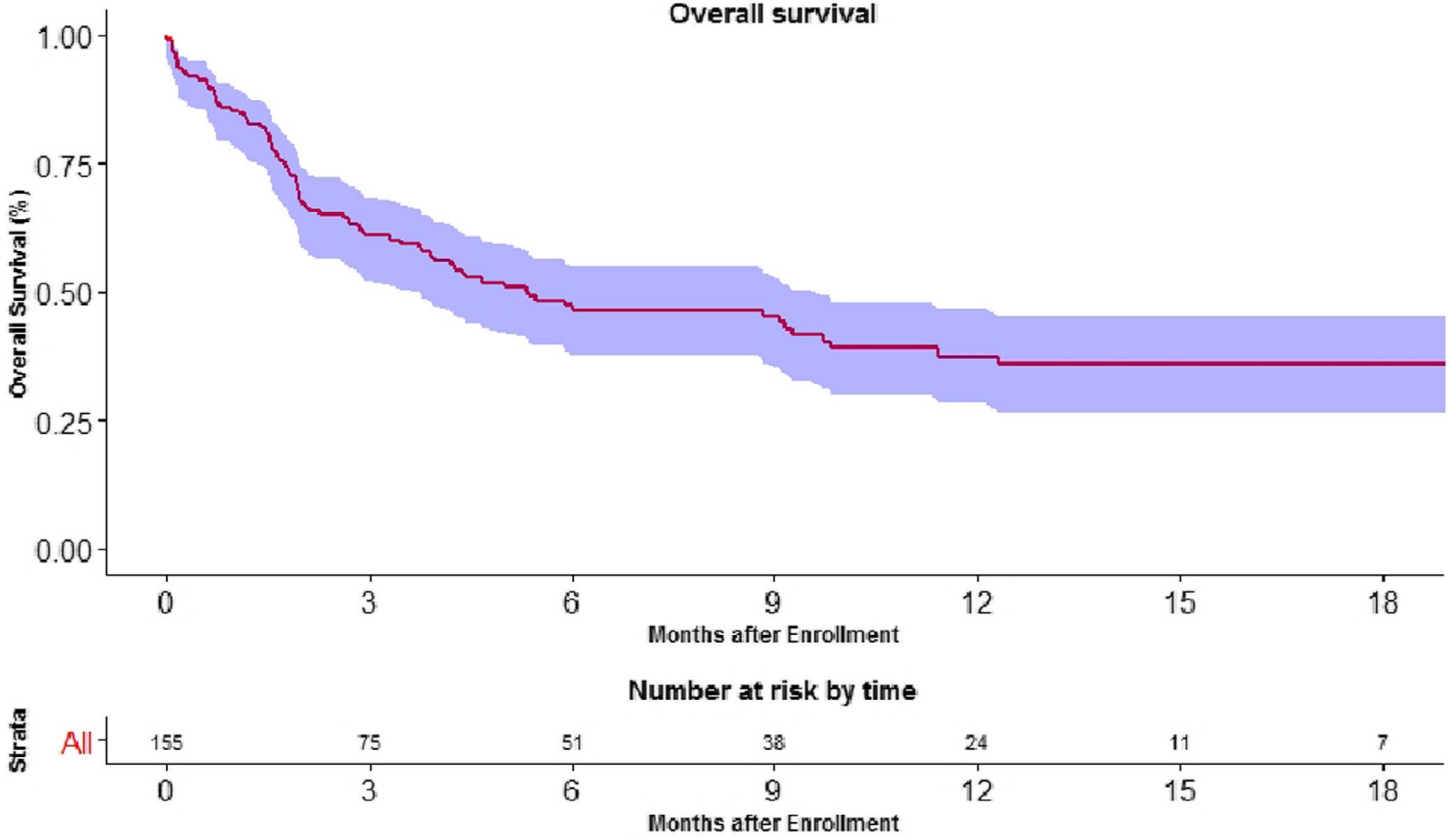
Kaplan Meier curve depicting overall survival in patients treated with ICI. The overall survival at 1 year was 37.5% (95% CI 28.4–46.6).

The median overall survival in non‐melanoma solid cancers was 5.37 months (95% CI, 3.73–9.73) (Figure 6).

### 4.4 ECOG performance status at baseline with progression free and overall survival

**FIGURE 7:**
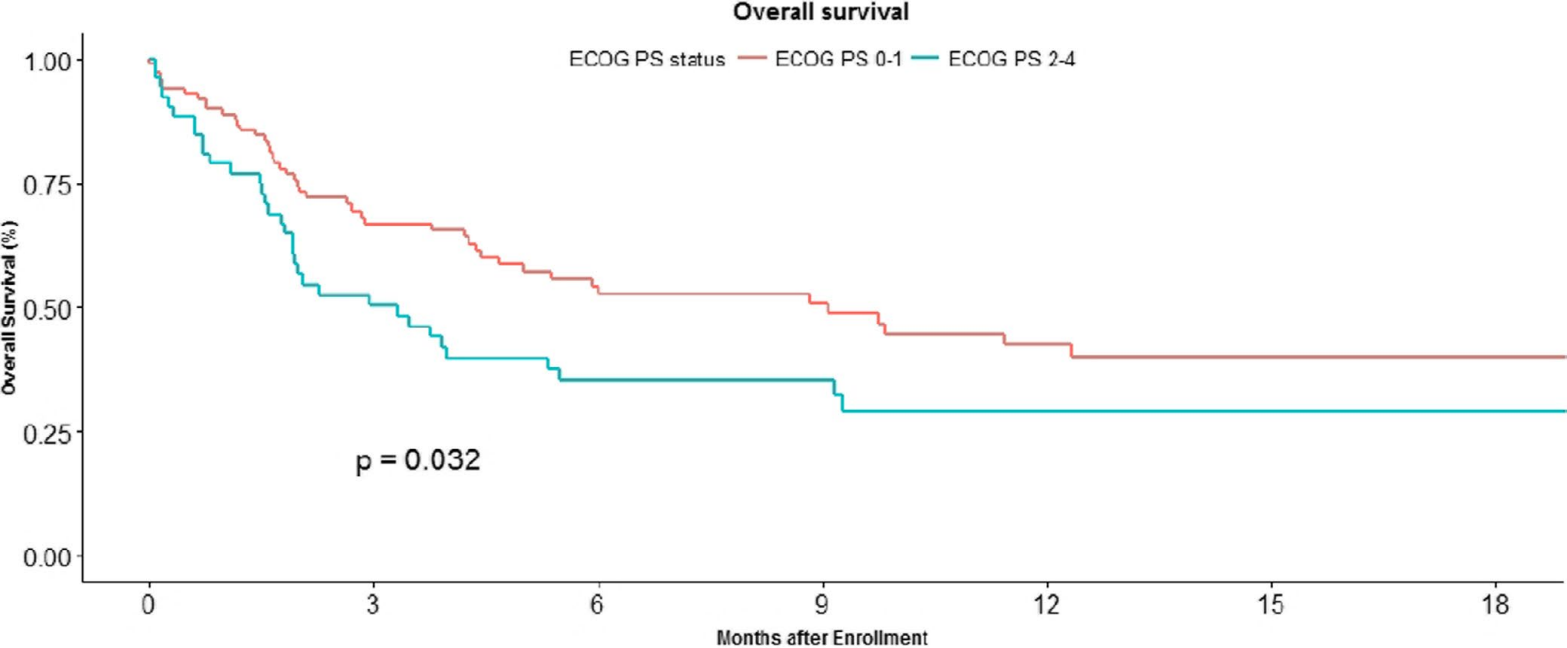
Kaplan Meier curve showing overall survival in patients on immune check point inhibitors comparing the baseline performance status ECOG‐1 was associated with median OS 9.07 (4.43‐NA) months when compared to ECOG 2‐3 which had a median OS of 3.30 (1.80–5.47) months (*p* = 0.032).

Univariate analysis was performed for overall survival taking gender, age, performance status, line of immunotherapy, addiction, diabetes as comorbid condition, use of steroids, use of antibiotics and site of primary tumor. Only performance status was a significant factor in this analysis. Baseline performance status ECOG‐1 is associated with median OS 9.07 (4.43‐NA) months when compared to ECOG 2–3 having a median OS of 3.30 (1.80–5.47) months (*p* = 0.032) (Figure 7). Thus, multivariate analysis could not be performed.

The performance status (PS) at baseline appeared to impact the progression free survival in lung cancer, with a numerical difference noted in the median PFS based on PS; median PFS was 3.5 months (95% CI, 1.41–5.59) in PS 1 patients versus 1.6 (95% CI, 1.34–1.85) months in patients with PS 2 and 3, *p* = 0.067. However, performance status at baseline did not impact PFS in head and neck cancer 1.8 (0.21–3.46) months in PS‐1 versus 1.9 (0.93–2.87) months in PS 2–3 *p* = 0.843.

**FIGURE 8:**
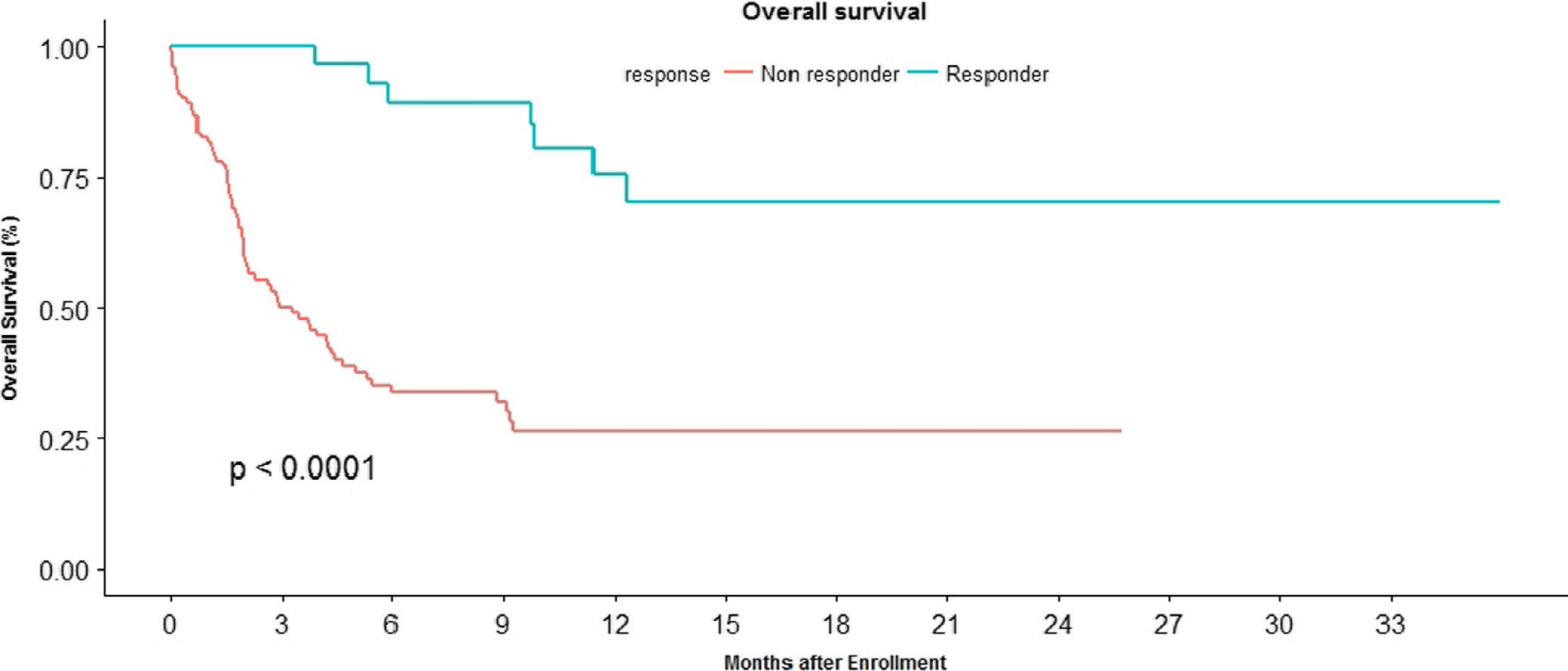
Kaplan Meier curve comparing the overall survival in ICI responders (blue) and ICI non responders (red). The median overall survival was not reached for responders of ICI at 30 months. The median overall survival for non responders of ICI was 3.3 (1.97–4.43) months. (*p* < 0.0001).

The median overall survival was not reached for responders of ICI at 30 months. The median overall survival for non‐responders of ICI was 3.3 (1.97– 4.43) months. (*p* < 0.0001) (Figure 8).

## 5 DISCUSSION

The use of immunotherapy has revolutionized the treatment of solid tumors in the last decade. There are limited Indian data on the safety and efficacy of immune checkpoint inhibitors for different solid tumors.^18^ Financial constraints and accessibility still remain as the strong barriers to the use of ICI in Indian patients.^19^

Real‐world data from our center confirm that the response rates of patients outside clinical trials in India are broadly similar to those reported in Western literature.^20^ The clinical benefit rate in lung cancer was 39.4% (*n* = 30/76) and response rate was 14.5% (*n* = 11/76) comparable to published literature on NSCLC.^21^ The clinical benefit rate and response rates in HNSCC was 33.3%(*n* = 16/48) and 14.5% (*n* = 7/48) similar to trial settings.^22^ The clinical benefit rate in genitourinary cancer including renal cell carcinoma was 52% (*n* = 13/25) but the numbers were small to draw definitive conclusions. This study also confirms that a significant proportion of patients who respond to treatment continue to have sustained response.^23^ This gives the hope for long‐term survival in these patients as reported in literature.^23^

The adverse events associated with ICI were rare and life‐threatening complications were infrequent facilitating their use in patients with poor performance status. There were no fatal toxicities noted. The cumulative incidence of adverse events with ICI was less than 10% which was comparable with the Western data.^24^

The use of antibiotics and steroids concomitantly with ICI was hypothesized to lead to a decreased efficacy of ICI.^25^, ^26^ Due to the small number of patients in our study, it is tough to make definitive conclusions but there was a trend towards worse outcome in patients receiving antibiotics although statistical significance was not reached. We were not able to get full details of these treatments due to retrospective nature of this study. There was no difference in overall survival based on steroid use along with ICI.

ECOG performance status is one of the most important prognostic factors in oncology. ECOG performance status did not determine survival with ICI as per available meta‐analysis data.^27^ In our study, ECOG performance status at baseline was an independent predictor of overall survival in patients who were initiated on ICI. Considering the relatively good safety profile of immunotherapeutic drugs, oncologists are tempted to use them even in poor performance status patients, but outcomes may be compromised. Our study confirms that ICIs can be administered safely in these patients. Some poor PS patients may respond to ICI.

Limitations of our study include single center recruitment of patients, PD‐LI (programmed cell death ligand 1) testing being done only in few patients *n* = 10/155 (6.4%) which is not feasible for analysis as an outcome determinant and selection bias due to less representation from general category (poor patients) due to lack of accessibility of ICI. Larger prospective studies are required in developing countries to understand the efficacy and long‐term safety of immune checkpoint inhibitors.

## 6 CONCLUSIONS

The real‐world data confirms similar benefit of immunotherapy as seen in trial settings. Adverse events rates are low but significant and different from what we have seen in chemotherapy. Financial constraint is still the main obstacle limiting the accessibility of immunotherapy in India.

## 7 DECLARATION

This manuscript has been read and approved by all the authors, the requirements for authorship have been met. We believe that the manuscript represents honest work and this information is not provided in another form.

## Data Availability

All authors agree to submit the data generated for the analysis of the original article‐ “A real world data of immune check point inhibitors in solid tumors from India” and shall be made available to the Journal Cancer Medicine on request.
